# Uncovering the molecular mechanism of *Gynostemma pentaphyllum* (Thunb.) Makino against breast cancer using network pharmacology and molecular docking

**DOI:** 10.1097/MD.0000000000032165

**Published:** 2022-12-09

**Authors:** Wen-Xiang Wang, Xiao-Yan He, Dong-Yang Yi, Xiao-Yan Tan, Li-Juan Wu, Ning Li, Bin-Bin Feng

**Affiliations:** a School of Pharmacy of Chongqing Three Gorges Medical College, Chongqing, China; b Chengdu University of Traditional Chinese Medicine, Chengdu, China.

**Keywords:** breast cancer, *Gynostemma pentaphyllum* (Thunb.) Makin, mechanism, molecular docking, network pharmacology

## Abstract

Because of their strong anti-cancer efficacy with fewer side effects, traditional Chinese medicines (TCM) have attracted considerable attention for their potential application in treating breast cancer (BC). However, knowledge about the underlying systematic mechanisms is scarce. *Gynostemma pentaphyllum* (Thunb.) Makino (GP), a creeping herb, has been regularly used as a TCM to prevent and treat tumors including BC. Again, mechanisms underlying its anti-BC properties have remained elusive. We used network pharmacology and molecular docking to explore the mechanistic details of GP against BC. The TCM systems pharmacology database and analysis platform and PharmMapper Server database were used to retrieve the chemical constituents and potential targets in GP. In addition, targets related to BC were identified using DrugBank and Therapeutic Target Database. Protein–protein interaction network, Gene Ontology, and Kyoto Encyclopedia of Genes and Genomes pathway enrichment analyses of crucial targets were performed using the Search Tool for the Retrieval of Interacting Genes/Proteins and database for annotation, visualization, and integrated discovery databases, whereas the network visualization analysis was performed using Cytoscape 3.8.2. In addition, the molecular docking technique was used to validate network pharmacology-based predictions. A comparison of the predicted targets of GP with those of BC-related drugs revealed 26 potential key targets related to the treatment of BC, among which ALB, EGFR, ESR1, AR, PGR, and HSP90AA1 were considered the major potential targets. Finally, network pharmacology-based prediction results were preliminarily verified by molecular docking experiments. In addition, chemical constituents and potential target proteins were scored, followed by a comparison with the ligands of the protein. We provide a network of pharmacology-based molecular mechanistic insights on the therapeutic action of GP against BC. We believe that our data will serve as a basis to conduct future studies and promote the clinical applications of GP.

## 1. Introduction

Breast cancer (BC), a highly common cancer reported in females, ranks second among cancer-related deaths in women globally. With a malignancy rate of 23%, BC has a mortality rate of 14%.^[1]^ Although the mainstay treatment includes chemotherapy, hormone therapy, surgery, and radiotherapy, these are associated with a considerable and long-term impact on patients.^[2]^ For instance, previous studies have reported the risk of acute myeloid leukemia among patients with BC undergoing radiotherapy and chemotherapy.^[3]^ Despite the great advances in modern cancer research medicine, BC has remained a major health concern due to its high rates of morbidity and mortality.^[4]^ Cisplatin, one of the most extensively used chemotherapeutic drugs in clinical studies against BC, is known to cause side effects, for example, nephrotoxicity; however, it is still extensively used owing to its high efficacy.^[5]^ Therefore, natural products with anti-cancer properties are increasingly being used to develop drugs to prevent and treat BC.^[6]^ Because several mechanisms, including cell proliferation inhibition of, enhanced cell apoptosis, and cell cycle arrest contribute to the inhibitory effects on BC,^[7]^ previous researchers have focused on several potential natural products to identify bioactive compounds for treating BC.

Traditional Chinese medicine (TCM) has emerged as an adjuvant treatment for cancer. TCM has been used to improve immune functions and the tumor microenvironment of patients and reduce the damage caused by radiotherapy and chemotherapy, thereby improving the survival rate.^[8]^ In addition, certain clinical research has reported that a combination of TCM and chemotherapy can not only reverse the drug resistance but also reduce the side effects of chemotherapy.^[9]^ *Gynostemma pentaphyllum* (Thunb.) Makino (*G. pentaphyllum*, GP), known as Jiaogulan, in Chinese, is a creeping herb that belongs to the Cucurbitaceae family, with major distribution in China. Moreover, it has been widely consumed as a dietary supplement and herbal tea since the Ming Dynasty.^[10]^ Herbal tea is also being increasingly used as a nutritional supplement even in North America and Europe.^[11]^ The physicians of ancient and modern have basically the same understanding of the etiology and pathogenesis of breast cancer, they believe that the mutual accumulation of phlegm, blood stasis and toxin is the key pathogenesis, so the first treatment is to soothe the liver and relieve depression, and at the same time, to strengthen the spleen and kidney through the whole process. To dispel evil, it is necessary to remove phlegm, blood stasis and toxin. In clinical practice, patients with malignant tumors often have symptoms such as fever, pain, local mass enlargement accompanied by redness, swelling, heat and pain. These symptoms, from the perspective of TCM theory, mostly belong to the syndrome of heat toxin accumulation. For malignant tumors caused by heat toxin, the cold drugs of heat-clearing and detoxifying should be used to eliminate heat toxin. According to the TCM theory, GP, a heat-clearing and detoxifying drugs in TCM, has a warm nature with a slightly bitter taste.^[12]^ A previous study demonstrated the use of GP to treat tumors, hematuria, edema, pain in the pharynx, and traum.^[13]^ In recent decades, multiple efficacies of GP, including anticancer, antimicrobial, antiaging, antifatigue, hypolipidemic, and immunoregulation,^[14]^ have been reported by pharmacological studies, which are attributed to its several chemical constituents, including saponins, flavonoids, polysaccharides, and trace elements.^[15]^

**Figure 1.**
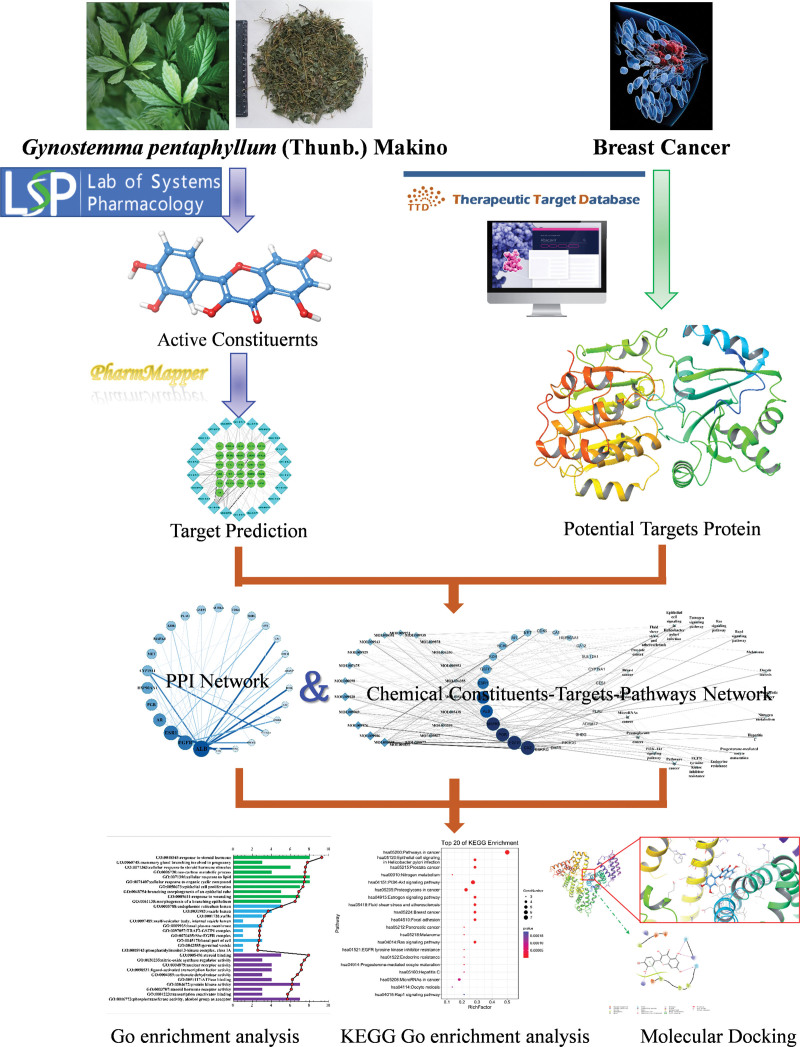
Study workflow.

Network pharmacology has emerged as a novel drug discovery approach that integrates systematic medicine with information science created by Hopkins in 2007.^[16]^ Currently, it is being used to study the association between drugs, their targets, and specific diseases, as well as discover and validate potential drug candidates.^[17]^ Molecular docking, a method to identify the binding sites and assess the binding affinity of drugs and target proteins, is a virtual screening method to uncover molecular interactions and predict their binding mode and affinity. Herein, we used a combination of molecular docking and network pharmacology to decipher the potential molecular anti-cancer mechanistic details of GP against BC (Fig. 1).

## 2. Materials and Methods

### 2.1. Chemical ingredient database building and active ingredient screening

We retrieved 202 compounds of GP using the data provided in published research papers and the public database (TCM Systems Pharmacology, https://www.tcmsp-e.com/). Their PubChem ID and two-dimensional chemical structures were retrieved from PubChem (https://pubchem.ncbi.nlm.nih.gov/). Their CAS numbers were obtained from the SciFinder database (https://scifinder.cas.org/). Next, we analyzed the physicochemical properties of the compounds of GP to identify the potentially active compounds. We used oral bioavailability ≥0.30 and drug-likeness ≥0.18 as criteria for screening active constituents.^[18]^

### 2.2. Target prediction

A significant component of a drug discovery program is whether active ingredients could interact with their specific targets.^[19]^ To assess this, drug discovery should aim at accurately identifying and validating drug–target interactions.^[20]^ For this, we retrieved the structure data file structure format of candidate compounds from PubChem Compound (https://www.ncbi.nlm.nih.gov/pccompound/). PharmMapper Server database (http://lilab.ecust.edu.cn/pharmmapper)^[21]^ was used with “*Homo sapiens*” species in the setting to identify the underlying targets linked to the selected constituents. Furthermore, the UniProt database (https://www.uniprot.org/) was used to retrieve the gene information. Therapeutic Target Database (TTD) (http://db.idrblab.net/ttd/) and DrugBank (https://www.drugbank.ca/) databases were used to search the targets on BC.^[22]^ Finally, Venn 2.1.0 (https://bioinfogp.cnb.csic.es/tools/venny/) was used to analyze common targets associated with GP and BC.

### 2.3. Network construction

We used Cytoscape 3.8.2, an open source for constructing and visualizing complex networks to construct a compound–target–pathway network and identify the relationships of target proteins with active ingredients and corresponding pathways.^[23]^ Next, an online STRING 11.5 (https://string-db.org/) database was used to construct a protein–protein interaction (PPI) network and analyze common targets between GP and BC.^[24]^ Afterward, we imported common targets into STRING 11.5 to study protein–protein interactions. Targets that were limited to the species “*Homo sapiens*” and had interaction scores ≥0.4 were used to identify interacting genes. The PPI network was studied using Cytoscape 3.8.2 and downloaded from the official website (https://cytoscape.org/).

### 2.4. Gene Ontology and Kyoto Encyclopedia of Genes and Genomes pathway enrichment analysis

Next, Gene Ontology (GO) and Kyoto Encyclopedia of Genes and Genomes (KEGG) pathway enrichment analyses were performed using the Database for Annotation, Visualization and Integrated Discovery (https://david.ncifcrf.gov/).^[25]^ The enrichment analysis revealed 3 GO categories, namely biological processes, cellular component, and molecular function, with a false discovery rate, corrected *P*-value < .05 (significant). In addition, the KEGG pathway analysis revealed the top 20 crucial pathways depending on the order of gene count and *P*-value (*P* < .05 were considered significant).

### 2.5. Molecular docking

**Figure 2.**
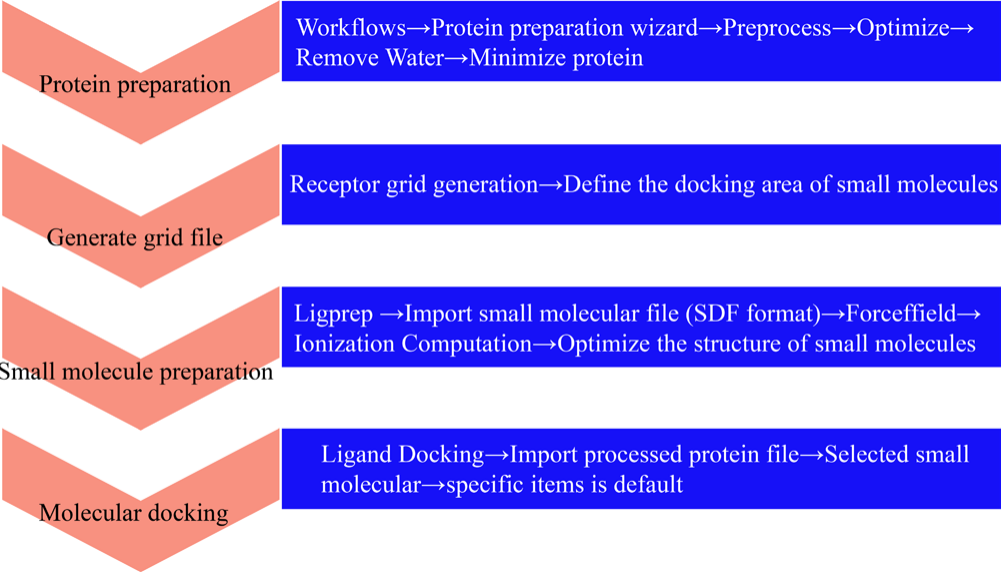
Molecular docking procedure.

The RCSB Protein Data Bank database (https://www.rcsb.org/) was used to retrieve the crystal structures of screened targets. In addition, their Protein Data Bank format files were downloaded. Files in the structure data file format of candidate compounds were downloaded from the PubChem database, following which these were uploaded to Maestro 11.1 to perform docking (Fig. 2) and obtain the docking score. The force field used in the energy minimization of the ligands is OPLS_2005, the determination of the binding pocket coordinates is generated with the protoligand of crystal structure as the center and the box size is set to 1 nm (10 Å), and other parameters are selected as the default values of the system, the module of Ligand Docking was used for molecular docking through standard precision. A score >4.25 indicated a small binding activity; a score >5 demonstrated a better binding activity, and that >7 represented a strong binding activity. The force field used in the energy minimization of the ligands is OPLS_2005. However, we decided to contrast with the ligand of the protein and took the docking pocket position of the ligand of the protein as the docking site, which increased the accuracy of the results of molecular docking experiments. All methods used were those described in published studies.^[26]^

## 3. Results

### 3.1. Active ingredient screening and potential targets of GP

We considered those compounds as active whose pharmacokinetic parameters met the following criteria: oral bioavailability ≥30% and drug-likeness ≥0.18.^[27]^ The detailed information of screening candidate compounds is shown in Table S1 (Supplemental Digital Content, http://links.lww.com/MD/I62, which illustrates the effective information of active compounds); and at length, 202 ingredients from GP remained 22. Traditional methods to identify protein targets of GP involve considerable manual efforts, resources, and cost However, the *in silico* model provides a rapid and efficient approach to identifying potential targets. Using the pharmacophore-matching method, and certain statistical and similarity measures, 108 protein targets were retrieved from the PharmMapper server database (see Table S2, Supplemental Digital Content, http://links.lww.com/MD/I63, which illustrates the potential target prediction of active ingredients).

### 3.2. Identification of BC protein targets

**Figure 3.**
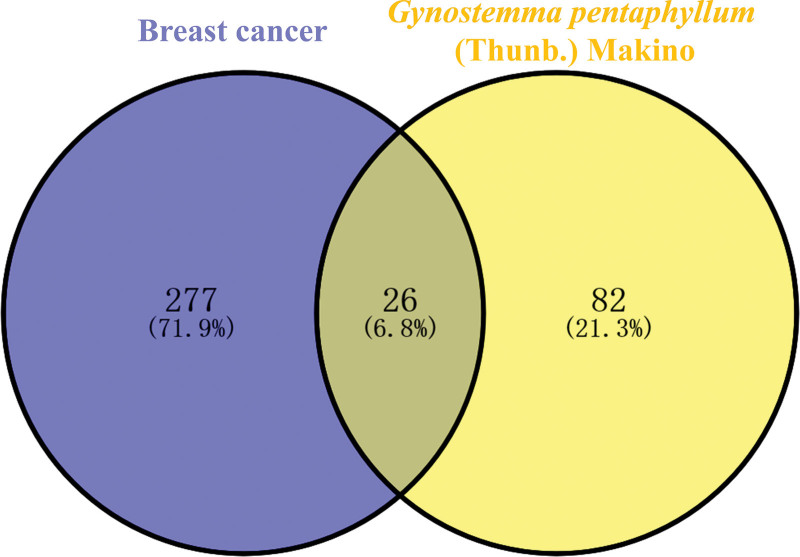
Venn diagram showing common targets of GP and BC. BC = breast cancer, GP = *Gynostemma pentaphyllum*.

After removing the duplicated items related to BC, retrieval results of DrugBank and TTD databases showed 309 genes (see Table S3, Supplemental Digital Content, http://links.lww.com/MD/I64, which illustrates the BC related targets in TTD and Drugbank database, 171 in DrugBank and 138 in TTD). The detailed information of common targets between BC and GP is listed in Table S4 (Supplemental Digital Content, http://links.lww.com/MD/I65, which illustrates the detailed information of common targets between BC and GP). Finally, 26 overlapping genes having potential therapeutic functions in GP against BC were identified using Venn 2.1.0 (Fig. 3).

### 3.3. GO and KEGG enrichment analyses of targets

**Figure 4.**
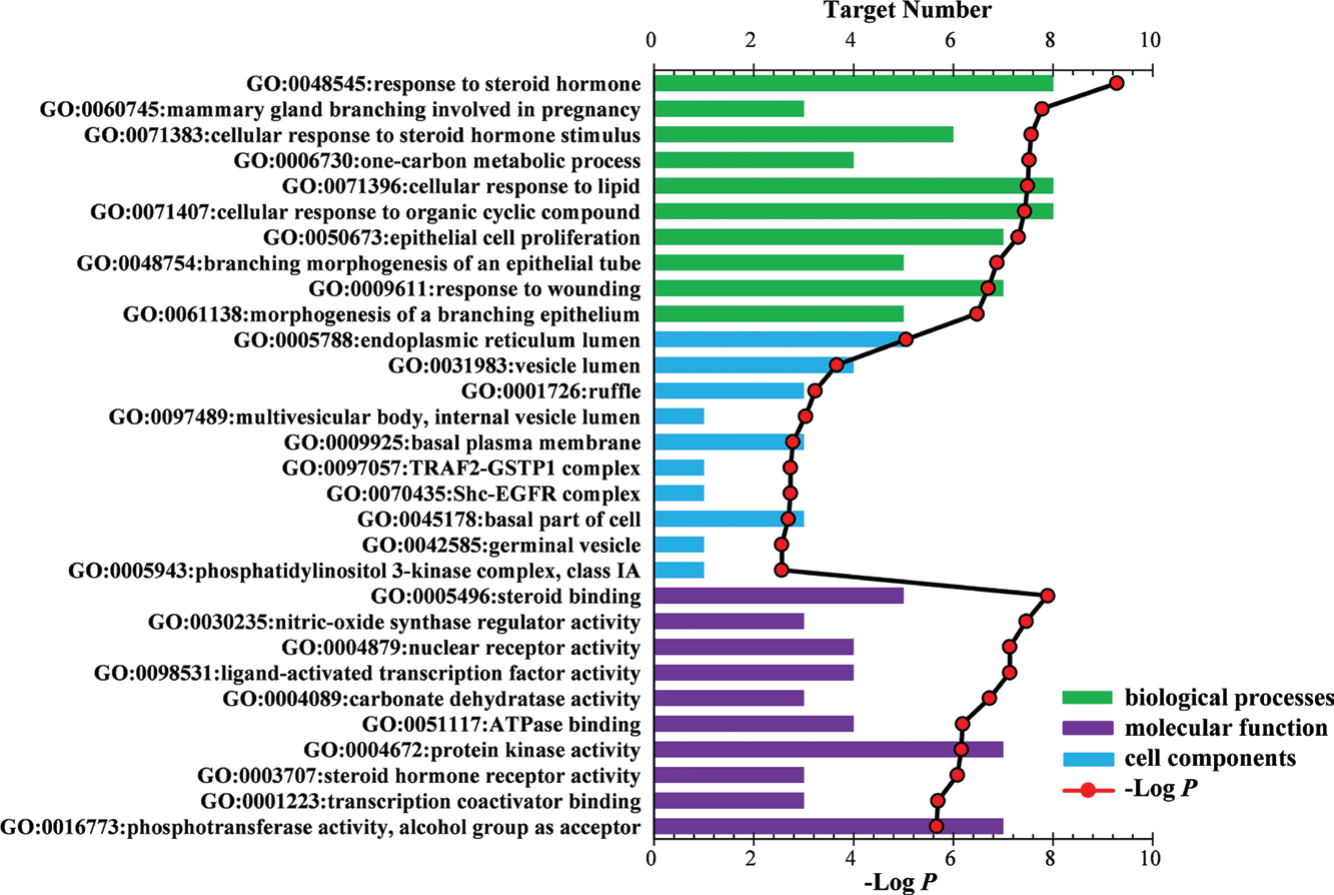
GO enrichment analysis results of biological processes, cell composition, and molecular function annotation. GO = gene ontology.

**Figure 5.**
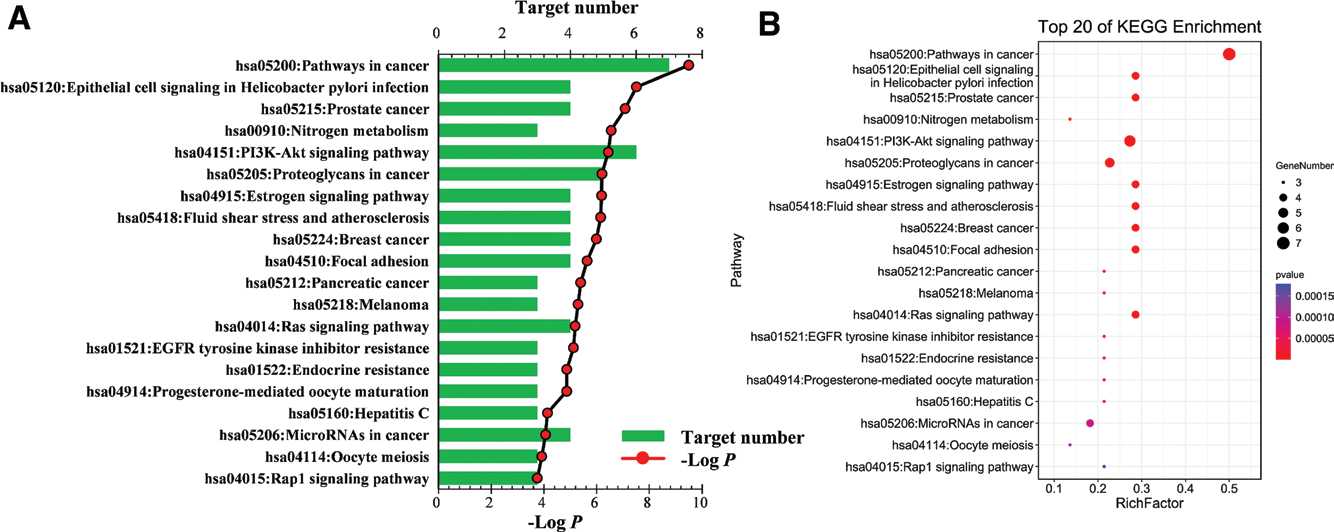
KEGG enrichment analysis of potential targets linked to the occurrence and development of BC. BC = breast cancer, KEGG = Encyclopedia of Genes and Genomes.

The top 10 and 20 entries of GO and KEGG enrichment analysis, respectively, of GP targets against BC were illustrated as histograms and a bubble chart, respectively (Figs. 4 and 5). Similarly, 90 GO entries were retrieved (Fig. 4 shows the top 10 according to *P*-value < .05); among these, 48 entries corresponded to biological processes, response to steroid hormones, mammary gland branching involved in pregnancy, cellular response to steroid hormone stimulus, one-carbon metabolism, cellular response to organic cyclic compounds, epithelial cell proliferation, branching morphogenesis of the epithelial tube, response to wounding, and morphogenesis of a branching epithelium. In addition, 27 items were related to molecular function, including steroid binding, nitric oxide synthase regulator activity, nuclear receptor activity, ligand-activated transcription factor activity, carbonate dehydratase activity, ATPase binding, protein kinase activity, steroid hormone receptor activity, transcription coactivator binding, phosphotransferase activity, and alcohol group as the acceptor. Furthermore, 15 cell component entries included endoplasmic reticulum lumen, vesicle lumen, ruffle, multivesicular body, internal vesicle lumen, basal plasma membrane, TRAF2–GSTP1 complex, Shc–EGFR complex, basal part of the cell, germinal vesicle, phosphatidylinositol 3-kinase complex, and class IA (see Table S5, Supplemental Digital Content, http://links.lww.com/MD/I66, which illustrates the Go and KEGG analysis of potential targets related to occurrence and development of BC).

Next, we determined the relationship between target proteins and biological pathways. Based on the KEGG analysis, we screened 25 pathways that corresponded to 26 protein targets (Fig. 5 shows the top 20 according to *P*-value < .05), including pathways in cancer, epithelial cell signaling during *Helicobacter pylori* infection, prostate cancer, nitrogen metabolism, PI3K–Akt signaling pathway, proteoglycans in cancer, estrogen signaling pathway, fluid shear stress and atherosclerosis, breast cancer, focal adhesion, and pancreatic cancer. We found that a single target protein was present in several pathways simultaneously, and several target proteins existed in a single pathway (see Table S5, Supplemental Digital Content, http://links.lww.com/MD/I66, which illustrates the Go and KEGG analysis of potential targets related to occurrence and development of BC).

These results demonstrated that the active components present in GP could serve as potential candidates for treating BC by regulating these signaling pathways.

### 3.4. PPI network construction

**Figure 6.**
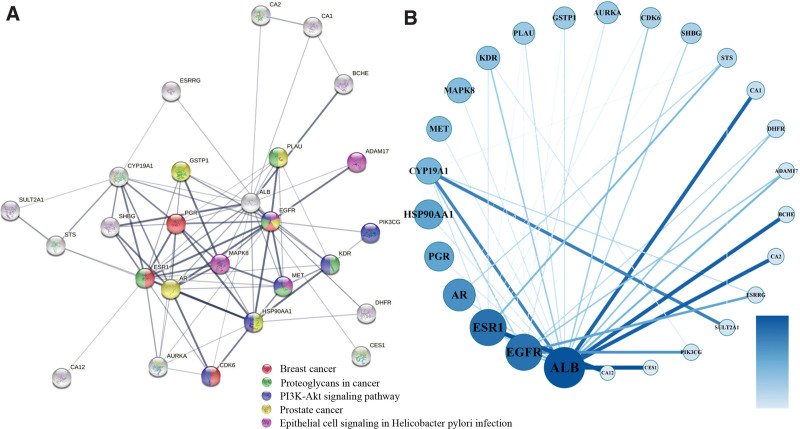
Protein–protein interaction network (A: constructed using String 11.0, the color of the node represented different signaling pathways as shown above; B constructed using Cytoscape 3.8.2, the node color had a positive association with the extent of its contribution to BC). BC = breast cancer.

We next constructed the PPI network (Fig. 6) of common target genes using STRING and Cytoscape 3.8.2 to elucidate their relationships. The nodes present in the PPI network reflect the interrelationships involved during the development of BC. The PPI network consisted of 26 nodes and 85 edges; the node color was positively related to the extent of its contribution to BC. The color of nodes in the PPI network such as *ALB*, *EGFR*, *ESR1*, *AR*, *PGR*, and *HSP90AA1* was darker and the corresponding degree values could be easily identified (Table 1) as 19, 16, 16, 13, 11, and 11. Based on these results, we believe that these could be crucial target genes in the development of BC.

**Table 1:** Degree values of crucial target proteins.

Target name	Gene name	UniProt ID	PDB ID	Betweenness centrality	Closeness centrality	Degree
Albumin	ALB	O60674	1HK4	0.377607	0.806452	19
Epidermal growth factor receptor	EGFR	Q02127	3POZ	0.172591	0.735294	16
Estrogen receptor	ESR1	P28482	1XPC	0.159369	0.735294	16
Androgen receptor	AR	P60568	1T65	0.052421	0.675676	13
Progesterone receptor	PGR	P01375	1SQN	0.020849	0.641026	11
Heat shock protein HSP 90-alpha	HSP90AA1	P35354	2YI7	0.025171	0.625	11

PDB = Protein Data Bank.

Above findings indicated *ALB*, *EGFR*, *ESR1*, *AR*, *PGR*, and *HSP90AA1* to be located at a key position and majorly involved in pathways related to cancer (has05200), PI3K–Akt signaling pathway (has04151), estrogen signaling pathways (hsa04915), breast cancer (hsa05224), EGFR tyrosine kinase inhibitor resistance (hsa01521), proteoglycans in cancer (hsa05205), and Ras signaling pathway (hsa04014), thereby suggesting their implication in regulating the occurrence and development of BC.

### 3.5. Compound–target–pathway network analysis of GP

**Figure 7.**
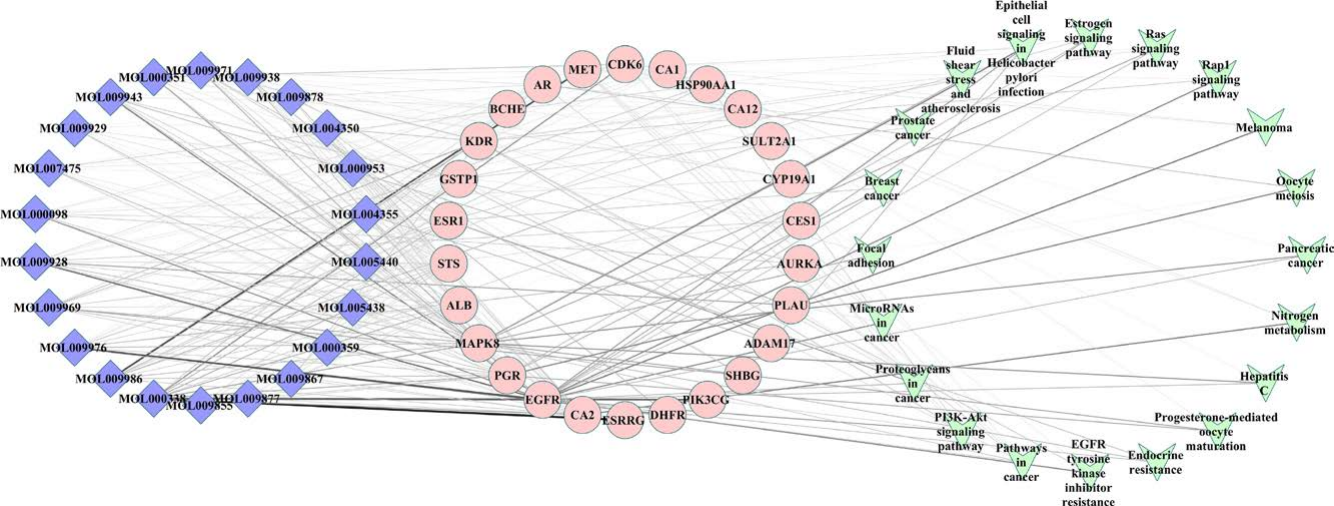
Network of component–target–pathway (the color of the node is positively related to the degree of its contribution to BC). BC = breast cancer.

KEGG pathway enrichment analysis was used to establish a component–target–pathway network and to further confirm the potential targets (Fig. 7). This network indicated that 22 compounds interacted with 26 targets and were associated with BC through 20 pathways. These 26 target proteins might exert a crucial impact on the development of BC. However, further animal and cellular studies, and clinical trials are necessary to confirm these speculations.

### 3.6. Molecular docking

**Figure 8.**
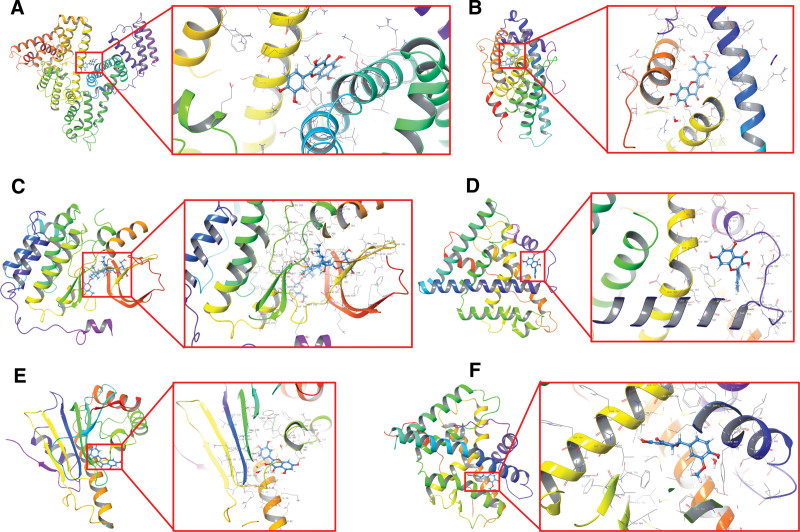
Molecular docking of active compounds and key targets (three-dimensional) (A: quercetin to ALB_1H4K; B: quercetin to AR_1T65; C: gypenoside XXXII to EGFR_3POZ; D: quercetin to ESR1_1XPC; E: quercetin to HSP90AA1_2YI7; F: homoeriodictyol to PGR_1SQN.).

**Figure 9.**
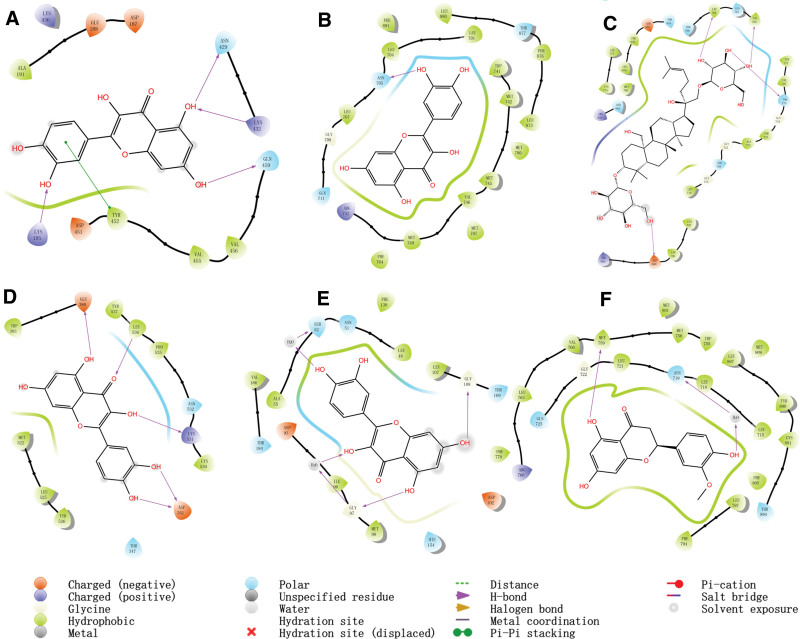
Molecular docking of active compounds and key targets (two-dimensional) (A: quercetin to ALB_1H4K; B: quercetin to AR_1T65; C: gypenoside XXXII to EGFR_3POZ; D: quercetin to ESR1_1XPC; E: quercetin to HSP90AA1_2YI7; F: homoeriodictyol to PGR_1SQN.).

For molecular docking, we selected 22 active ingredients with 6 pivotal target proteins using the procedure shown in Figure 2. The score of the ligand of the target proteins was named “contrast.” The compound (docked with target proteins) whose score was close to or greater than this contrast value was considered as an active compound acting on the target protein. The top 3 compounds bound to the crucial target proteins are summarized in Table 2, and the highest-scoring compound and ligand with target proteins are shown in Figure 8 (three-dimensional) and Figure 9 (two-dimensional).

**Table 2:** Docking scores of the top 3 compounds bound to crucial target proteins.

Proteins	Compounds	Glide gscore	Glide hbond	Glide evdw	Glide ecoul	Glide energy
ALB (1H4K)	Contrast	−6.105	−0.649	−33.527	−9.973	−43.501
	Quercetin	−6.976	−0.116	−32.582	−8.802	−41.384
	Homoeriodictyol	−6.536	0.000	−33.049	−5.201	−38.250
	Rhamnazin	−6.639	−0.037	−35.771	−6.242	−42.013
AR (1T65)	Contrast	−10.064	0	−43.826	−3.041	−46.867
	Quercetin	−9.159	−0.305	−32.884	−9.207	−42.091
	Rhamnazin	−7.399	−0.191	−27.877	0.737	−27.140
	Homoeriodictyol	−9.054	−0.509	−32.465	−6.108	−38.573
EGFR (3POZ)	Contrast	−12.931	−0.528	0.000	−2.638	−64.991
	Quercetin	−7.462	−0.011	−40.373	−4.691	−45.065
	Rhamnazin	−6.983	0.000	−35.781	−4.054	−39.835
	Gypenoside XXXII	−8.650	−0.304	−35.785	−20.862	−56.647
ESR1 (1XPC)	Contrast	−12.861	−0.737	0.000	−1.366	−60.120
	Quercetin	−6.521	0	−22.876	−1.874	−24.750
	Homoeriodictyol	−5.587	−0.182	−23.229	−4.451	−27.680
	Rhamnazin	−6.062	−0.037	−25.257	−2.302	−27.559
HSP90AA1 (2YI7)	Contrast	−9.146	−0.469	0.000	−1.030	−44.180
	Quercetin	−8.571	−0.320	−28.626	−15.278	0.202
	Homoeriodictyol	−8.030	−0.144	−30.367	−12.938	0.201
	Gypenoside LXXIV	−6.942	−0.039	−31.347	−13.344	0.187
PGR (1SQN)	Contrast	−9.467	−0.094	0.000	−2.724	−38.126
	Homoeriodictyol	−9.387	0.000	0.000	−2.449	−31.304
	Quercetin	−8.822	0.000	0.000	−2.141	−37.882
	Rhamnazin	−6.411	0.000	0.000	−0.001	−34.635

Glide hbond refers to hydrogen bonding. Glide evdw refers to Van der Waals interactions. Glide ecoul refers to Coulomb energy. All items are components the Glide gscore algorithm.

## 4. Discussion

Factors such as lack of absorption, distribution, metabolism, and excretion properties of drugs make it extremely difficult to identify and develop novel drug candidates. Therefore, researchers have diverted their attention toward efficient absorption, distribution, metabolism, and excretion-based screening.^[28]^ In this regard, network pharmacology, an intersectional discipline including classical pharmacology, molecular biology, and bioinformatics, is highly effective in identifying potential targets and pathways and drug–target interactions.^[29]^ In addition, network pharmacology can be used to study complexities among drugs, target proteins, and diseases.^[30]^ Virtual drug screening using molecular docking has emerged as a highly significant component of structural biology. Therefore, a combination of molecular docking and network pharmacology can efficiently predict potential drug–target relationships, thereby contributing to drug discoveries in complicated diseases such as BC. Here, we predicted the potential mechanism between GP and BC by integrating the relative information obtained from publicly available databases.

BC is regarded as one of the commonest cancers affecting women’s health worldwide.^[31]^ TCM has gained increasing attention among researchers to treat BC due to it minor side effects and good therapeutic effects. The mechanism of action of TCM against BC involves reverting drug resistance, inhibiting metastasis and proliferation, inducing tumor cell apoptosis, and arresting the cell cycle.^[32]^

GP (*G. pentaphyllum*), referred to as “Jiao-Gu-Lan” in China, belongs to the family Cucurbitaceae.^[33]^ With an abundance of presence in Asia, it has been used in food and vegetables for hundreds of years, particularly in the south of the Qinling Mountains and Yangtze River of China.^[34]^ GP has several therapeutic properties; for example, according to the TCM theory, it can regulate qi flowing, reinforce qi, and nourish the blood. Our data mining results were found to be consistent with the above findings on the pathogenesis of BC.^[12]^ Best known as a herbal remedy, GP has been used to effectively treat multiple diseases because of its anti-cancer^[35]^ and anti-inflammatory effects.^[36]^ The pathogenesis of BC has been reported to involve qi stagnation and deficiency of qi and blood. Although clinically, GP has been largely prescribed for breast hyperplasia and BC, its active ingredients and mechanism against BC have remained elusive. We used a combination of network pharmacology and molecular docking techniques to study the active compounds and mechanism of action of GP.

In total, 22 active compounds of GP, including quercetin and rhamnazin, were screened from previous research. Quercetin is regarded as an anti-cancer compound due to its strong anti-proliferation, apoptotic, and cell cycle-arresting properties.^[37]^ PhamMapper server, DrugBank, and TTD databases were used to identify the target genes that predicted the potential targets compounds in GP. Finally, BC-related targets were selected.

The GO enrichment analysis showed enrichment of common target genes to the endoplasmic reticulum (GO:0005788), vesicle (GO:0031983), and basal plasma membrane (GO:0009925). In addition, these largely correlated with TRAF2–GSTP1 complex (GO:0097057), Shc–EGFR complex (GO:0070435), basal part of the cell (GO:0045178), germinal vesicle (GO:0042585), phosphatidylinositol-3-kinase complex, and class IA (GO:0005943), indicating their participation in several cell metabolic reactions. In relation to biological processes, these genes majorly corresponded to the response to steroid hormones (GO:0048545), mammary gland branching involved in pregnancy (GO:0060745), cellular response to steroid hormone stimulus (GO:0071383), one-carbon metabolism (GO:0006730), cellular response to lipids (GO:0071396), epithelial cell proliferation (GO:0050673), and branching morphogenesis of the epithelial tube (GO:004875), which could be associated with cell apoptosis. With regard to molecular function, we found that these genes corresponded to steroid binding (GO:0005496), nitric oxide synthase regulator activity (GO:0030235), nuclear receptor activity (GO:0004879), ligand-activated transcription factor activity (GO:0098531), carbonate dehydratase activity (GO:0004089), ATPase binding (GO:0051117), protein kinase activity (GO:0004672), steroid hormone receptor activity (GO:0003707), and transcription coactivator binding (GO:0001223). These findings suggested that GP exerts its therapeutic potential against GP by interfering with the above-mentioned metabolic reactions and pathways.

Next, the KEGG enrichment analysis implicated several target genes of GP in cancer-related pathways (hsa05200), PI3K–Akt signaling pathway (hsa04151), estrogen signaling pathway (hsa04915), breast cancer (hsa05224), EGFR tyrosine kinase inhibitor resistance (hsa01521), proteoglycans in cancer (hsa05205), and Ras signaling pathway (hsa04014). Thus, GP displayed its anti-BC effects by regulating these pathways. The pathogenesis of BC is a highly complicated process because several factors are known to increase the risk of BC by interacting with a multitude of immune molecules and hormones. For instance, Beatson reported a connection between estrogen and BC in 1986.^[38]^ Similarly, further studies proved that estrogen binds to ERs and directly interacts with key signaling molecules (Shc) and membrane receptors (such as EGFR, ESR1, and IGFR) to activate the PI3K/AKT pathways, thereby promoting the proliferation and survival of tumor cells,^[39]^ The Shc–EGFR complex (GO:0070435) revealed by GO enrichment analysis in our study supports this finding. The PI3K–Akt pathway is a highly crucial pathway in cancer development,^[40]^ with its alterations observed in more than 70% of breast tumors; thus, it represents a significant therapeutic target against BC.^[41]^ Therefore, one of the potential therapeutic strategies against BC is to inhibit the estrogen signaling pathway (estrogen production blockage or ER function inhibition). Similarly, proteoglycans, crucial molecular effectors at the cell pericellular microenvironments of BC, undergo modification during tumorigenesis.^[42]^ Because proteoglycans control a variety of metabolic processes, they reveal novel therapeutic modalities against cancers. For instance, compared with normal breast tissues, previous research revealed varying expression of proteoglycans in malignant tissues. Furthermore, a previous study reported the therapeutic effect of inhibiting proteoglycans against BC. Another molecular aspect considered vital in this network is EGFR tyrosine kinase inhibitor resistance.^[43]^ EGFR is a transmembrane receptor tyrosine kinase that functions as a crucial component of cell signaling pathways.^[44]^ Clinically, tyrosine kinase inhibitors are used to treat cancers having *EGFR* mutations or their aberrant activation.^[45]^ Because *EGFR* mutations are known to induce drug resistance, researchers have used miRNAs to treat cancers displaying resistance toward EGFR– tyrosine kinase inhibitors.^[46]^

The results of PPI demonstrated 6 pivotal genes (*ALB*, *EGFR*, *ESR1*, *AR*, *PGR*, and *HSP90AA1*) as significant contributors to treating BC using GP because of the high betweenness centrality and degree values. In addition, increasing evidence has supported this opinion, demonstrating the participation of these genes in the pathophysiology and treatment of BC. Estrogen receptor 1 (ESR1), an estrogen receptor regulating mammary gland growth and differentiation, has been implicated in the pathological processes of BC. It binds to estrogen to promote the proliferation and differentiation of breast cells.^[47]^ Furthermore, studies have uncovered that excessive endogenous estrogen results in pathological changes in BC cells, thus a genetic variation in ESR1 could increase the potential risk of BC. Furthermore, progesterone has been reported to participate in the development of BC; Additionally, progesterone also served an important effect in patients with estrogen receptor and progesterone receptor (PGR), the prevalence of BC is less than 10%.^[48]^ Androgen receptor (AR), a nuclear hormone receptor, is highly expressed in BC, making it a strong therapeutic target.^[49]^ In addition, AR belongs to the steroid receptor family and has emerged as a considerable prognostic marker and underlying therapeutic target for BC.^[50]^ Similarly, HSP90AA1 is a common heat shock protein that promotes and maintains the function of intracellular proteins involved in apoptosis and the cell cycle.^[51]^ HSP90AA1 is known to maintain the stability of estrogen receptor, PGR, HER2, and relative downstream proteins.^[52]^ For example, overexpressed HSP90AA1 is related to poor prognosis in BC, suggesting it to be a valid therapeutic target. Moreover, HSP90AA1 is known to stabilize several proteins, with upregulated HSP90AA1 known to enhance the effectiveness of clinical intervention in BC.^[53]^ Another molecule is PGR which is known to regulate the progesterone genomic pathways; ER/PGR positivity has been widely used as a potential treatment target in BC. Over 80% of patients with BC are known to be ESR1-positive, with mutations in *ESR1* related to the resistance to aromatase inhibitor treatment. EGFR, an epidermal growth factor receptor, is known to induce proliferation in cancer and is overexpressed in 15% to 30% of patients with BC.^[54]^ It is reported that the drug induces apoptosis in glioblastoma through EGFR-activated Akt/MAPK signaling pathway.^[55]^ Therefore, targeted drugs against EGFR are known to reduce tumor progression and metastasis in patients with BC. Altogether, numerous previous studies support the findings of the present study.

Among the 6 crucial targets used for molecular docking experiments, EGFR, ESR1, PGR, and HSP90AA1 were identified as protein targets in the PI3K–Akt signaling and estrogen signaling pathways. Quercetin and rhamnazin were successfully docked with these 6 target proteins, with higher scores compared with others. In addition, molecular docking showed that 3 of the higher active ingredients had higher or closed scores than the positive control drugs (quercetin, rhamnazin, and homoeriodictyol). Several studies have reported the use of flavonoids for preventing and treating BC, making it indispensable to probe their potential in cancer treatments. Quercetin, a kind of flavonoid present in several plants, hinders the proliferation and promote the apoptosis of BC cells.^[56]^ Although quercetin is known to stimulate apoptosis, it inhibits cell processes by regulating vital BC-associated pathways, including PI3K–Akt, EGFR, estrogen, and MAPK signaling pathways.^[57]^ Moreover, quercetin can enhance the susceptibility of BC cells to several anticancer drugs, including docetaxel, cisplatin, topotecan, tamoxifen, sorafenib, rapamycin, paclitaxel, lapatinib, doxorubicin, and vincristine.^[58,59]^ Rhamnazin, a kind of flavonoid, directly acts against BC cells by inhibiting the proliferation, migration, and tube formation of HUVECs in vitro.^[60]^ In addition, homoeriodictyol is known to upregulate the expression of *p53*, *caspase-3*, and *Bax* genes, providing novel insights into its comparative anticancer efficacy and suggesting its underlying clinical applications as anticancer agents.^[61]^ These findings validated the active components screened by network pharmacology and their interactions with BC targets. However, little is known about their effects on BC. The above results suggested the potential of these compounds as anticancer agents. In addition, these previous studies supported that GP could work against BC by regulating the above-mentioned pivotal genes.

## 5. Conclusion

We studied and identified the active ingredients and molecular mechanism of GP against BC using a pharmacology network approach. The results demonstrated the underlying therapeutic effects of quercetin, rhamnazin, and homoeriodictyol on BC. In addition, we found that TCM provides a better therapeutic effect when used in combination with these compounds. Furthermore, the effective anti-cancer ingredients in GP can be probed for the underlying pharmacological mechanism against BC. We found that *ALB*, *EGFR*, *ESR1*, *AR*, *PGR*, and *HSP90AA1* exerted a significant effect in GP against BC. We believe that the current findings will inspire and guide researchers to conduct further work to validate the crucial targets of GP against BC for its clinical applications. Altogether, network pharmacology can provide new insights to explore TCM. A combination of network pharmacology and molecular docking is an efficient strategy and a convenient tool to study active components and mechanisms of TCM against multiple targets. However, there were some limitations to this study. For example, although there are some clinical studies on the anti-cancer and immunomodulatory effects of the compounds analyzed, these mechanisms need to be further verified. Although network pharmacology is an efficient method for predicting drug targets in sophisticated diseases, it is still necessary to verify the scientific nature and rationality of predicted targets by in vivo and in vitro experiments.

## Author contributions

**Conceptualization:** Wenxiang Wang, Ning Li, Binbin Feng.

**Data curation:** Wenxiang Wang, Xiaoyan He.

**Funding acquisition:** Wenxiang Wang, Lijuan Wu, Xiaoyan Tan, Binbin Feng.

**Supervision:** Dongyang Yi, Ning Li.

**Visualization:** Wenxiang Wang, Xiaoyan He.

**Writing – original draft:** Wenxiang Wang.

**Writing – review & editing:** Ning Li, Binbin Feng.

## Supplementary Material


