# Supplementary material for: Uncovering the molecular mechanism of Gynostemma pentaphyllum (Thunb.) Makino against breast cancer using network pharmacology and molecular docking
Source: Medicine (Baltimore). 2022 Dec 9;101(49):e32165. doi: 10.1097/MD.0000000000032165 (PMC9750687; doi:10.1097/MD.0000000000032165)
Supplement: Supplementary file 1 [file medi-101-e32165-s001.pdf]

Table S1 Effective information of active compounds

Mol ID	Molecule Name	CAS No.	Molecular Formula	MW	AlogP	Hdon	Hacc	OB (%)	DL (%)
MOL000338	Homoeriodictyol	446-71-9	$C_{16}H_{14}O_6$	302.3	2.28	3	6	51.61	0.27
MOL000351	Rhamnazin	552-54-5	$C_{17}H_{14}O_7$	330.31	2.01	3	7	47.14	0.34
MOL000359	sitosterol	83-46-5	$C_{29}H_{50}O$	414.79	8.08	1	1	36.91	0.75
MOL004350	Coroglaucigenin	468-19-9	$C_{23}H_{34}O_5$	390.57	2.29	3	5	36.12	0.76
MOL004355	Spinasterol	481-18-5	$C_{29}H_{48}O$	412.77	7.64	1	1	42.98	0.76
MOL005438	campesterol	474-62-4	$C_{28}H_{48}O$	400.76	7.63	1	1	37.58	0.71
MOL005440	Isofucosterol	18472-36-1	$C_{29}H_{48}O$	412.77	7.83	1	1	43.78	0.76
MOL007475	ginsenoside F2	62025-49-4	$C_{42}H_{72}O_{13}$	785.14	2.3	9	13	36.43	0.25
MOL000953	cholesterol	80356-14-5	$C_{27}H_{46}O$	386.73	7.38	1	1	37.87	0.68
MOL000098	quercetin	117-39-5	$C_{15}H_{10}O_7$	302.25	1.5	5	7	46.43	0.28
MOL009855	(24S)-Ethylcholesta-5,22,25-trans-3beta-ol	26315-07-1	$C_{29}H_{46}O$	410.75	7.44	1	1	46.91	0.76
MOL009867	4α,14α-dimethyl-5α-ergosta-7,9(11),24(28)-trien-3β-ol	128478-66-0	$C_{30}H_{48}O$	424.78	7.71	1	1	46.29	0.76
MOL009877	cucurbita-5,24-dienol	35012-08-9	$C_{30}H_{50}O$	426.8	7.93	1	1	44.02	0.74
MOL009878	Cyclobuxine	2241-90-9	$C_{25}H_{42}N_2O$	386.69	2.89	3	3	84.48	0.7
MOL009928	Gypenoside LXXIV	110261-97-7	$C_{42}H_{72}O_{14}$	801.14	1.21	10	14	34.21	0.24
MOL009929	Gypenoside LXXIX	110282-46-7	$C_{42}H_{72}O_{13}$	785.14	2.38	9	13	37.75	0.25
MOL009938	Gypenoside XII	62025-49-4	$C_{42}H_{72}O_{13}$	785.14	2.3	9	13	36.43	0.25
MOL009943	Gypenoside XL	90058-57-4	$C_{42}H_{70}O_{14}$	799.12	1.46	9	14	30.89	0.21
MOL009969	Gypenoside XXXV_qt	86589-62-0	$C_{30}H_{50}O_4$	444.77	5.92	2	3	37.73	0.78

MOL009971	20(S)-dammar-24-ene-3β,19,20-triol	208997-27-7	$C_{30}H_{52}O_3$	418.73	5.07	3	3	30.21	0.74
MOL009976	Gypenoside XXXII	90058-52-9	$C_{42}H_{72}O_{14}$	801.02	1.39	10	14	34.24	0.25
MOL009986	3β,20(S)-dihydroxydammar-24-en-12,23-dione	2416757-76-9	$C_{30}H_{48}O_4$	472.78	4.6	2	4	36.13	0.8
