# Supplementary material for: Uncovering the molecular mechanism of Gynostemma pentaphyllum (Thunb.) Makino against breast cancer using network pharmacology and molecular docking
Source: Medicine (Baltimore). 2022 Dec 9;101(49):e32165. doi: 10.1097/MD.0000000000032165 (PMC9750687; doi:10.1097/MD.0000000000032165)
Supplement: Supplementary file 2 [file medi-101-e32165-s002.pdf]

Table S2 Potential target prediction of active ingredients

Protein names	Uniprot ID	Gene names	Length
Steryl-sulfatase	P08842	STS	583
Albumin	P02768	ALB	609
Bone morphogenetic protein 2	P12643	BMP2	396
Transthyretin	P02766	TTR	147
Mitogen-activated protein kinase 10	P53779	MAPK10	464
Glutathione S-transferase P	P09211	GSTP1	210
S-methyl-5'-thioadenosine phosphorylase	Q13126	MTAP	283
Carbonic anhydrase 2	P00918	CA2	260
Aldo-keto reductase family 1 member B1	P15121	AKR1B1	316
Mitogen-activated protein kinase 8	P45983	MAPK8	427
Caspase-7	P55210	CASP7	303
Tyrosine-protein kinase HCK	P08631	HCK	526
ADP-ribose pyrophosphatase, mitochondrial	Q9BW91	NUDT9	350
Aldo-keto reductase family 1 member C2	P52895	AKR1C2	323
Apolipoprotein A-II	P02652	APOA2	100
Kinesin-like protein KIF11	P52732	KIF11	1056
Methionine aminopeptidase 1	P53582	METAP1	386
Peptidyl-prolyl cis-trans isomerase A	P62937	PPIA	165
Collagenase 3	P45452	MMP13	471
Cholinesterase	P06276	BCHE	602
Estradiol 17-beta-dehydrogenase 11	Q8NBQ5	HSD17B11	300
MAP kinase-activated protein kinase 2	P49137	MAPKAPK2	400
Probable ATP-dependent RNA helicase DDX6	P26196	DDX6	483
Sulfotransferase 2A1	Q06520	SULT2A1	285
Amine oxidase [flavin-containing] B	P27338	MAOB	520
Complement factor B	P00751	CFB	764
Thyroid hormone receptor beta	P10828	THRB	461
TGF-beta receptor type-2	P37173	TGFBR2	567
Galectin-10	Q05315	CLC	142
Serine/threonine-protein kinase Chk1	O14757	CHEK1	476
Carbonic anhydrase 1	P00915	CA1	261
Prolyl endopeptidase FAP	Q12884	FAP	760
Ephrin type-B receptor 4	P54760	EPHB4	987
Cathepsin D	P07339	CTSD	412
Lysosomal acid glucosylceramidase	P04062	GBA	536
Heat shock protein HSP 90-alpha	P07900	HSP90AA1	732
Troponin C, slow skeletal and cardiac muscles	P63316	TNNC1	161
Vitamin D-binding protein	P02774	GC	474

Stromelysin-1	P08254	MMP3	477
Progesterone receptor	P06401	PGR	933
Cathepsin L2	O60911	CTSV	334
Peroxisome proliferator-activated receptor gamma	P37231	PPARG	505
Carbonic anhydrase 12	O43570	CA12	354
Mitogen-activated protein kinase 1	P28482	MAPK1	360
Triggering receptor expressed on myeloid cells 1	Q9NP99	TREM1	234
17-beta-hydroxysteroid dehydrogenase type 1	P14061	HSD17B1	328
Liver carboxylesterase 1	P23141	CES1	567
cAMP-specific 3',5'-cyclic phosphodiesterase 4B	Q07343	PDE4B	736
Caspase-3	P42574	CASP3	277
Cyclin-A2	P20248	CCNA2	432
Epidermal growth factor receptor	P00533	EGFR	1210
Cathepsin S	P25774	CTSS	331
Cyclin-dependent kinase 2	P24941	CDK2	298
Urokinase-type plasminogen activator	P00749	PLAU	431
Androgen receptor	P10275	AR	920
Glycogen phosphorylase, liver form	P06737	PYGL	847
Chymase	P23946	CMA1	247
Heat shock cognate 71 kDa protein	P11142	HSPA8	646
Estrogen receptor	P03372	ESR1	595
Atrial natriuretic peptide receptor 3	P17342	NPR3	541
Purine nucleoside phosphorylase	P00491	PNP	289
Integrin alpha-L	P20701	ITGAL	1170
Hexokinase-1	P19367	HK1	917
Lanosterol synthase	P48449	LSS	732
Coagulation factor X	P00742	F10	488
Bone morphogenetic protein 7	P18075	BMP7	431
Serine/threonine-protein kinase pim-1	P11309	PIM1	313
Peptidyl-prolyl cis-trans isomerase FKBP1A	P62942	FKBP1A	108
Dihydrofolate reductase	P00374	DHFR	187
Oxysterols receptor LXR-beta	P55055	NR1H2	460
Prothrombin	P00734	F2	622
Alpha-amylase 1B (EC 3.2.1.1)	P0DTE7	AMY1B	511
Alpha-amylase 1C (EC 3.2.1.1)	P0DTE8	AMY1C	511
Alpha-amylase 1A (EC 3.2.1.1)	P0DUB6	AMY1A	511
Mitogen-activated protein kinase 14	Q16539	MAPK14	360
Glutaminyl-peptide cyclotransferase	Q16769	QPCT	361
Inositol monophosphatase 1	P29218	IMPA1	277

cGMP-specific 3',5'-cyclic phosphodiesterase	O76074	PDE5A	875
Aurora kinase A	O14965	AURKA	403
Beta-secretase 1	P56817	BACE1	501
Immunoglobulin alpha Fc receptor	P24071	FCAR	287
Nuclear receptor ROR-alpha	P35398	RORA	523
ATP-dependent Clp protease proteolytic subunit, mitochondrial	Q16740	CLPP	277
Aromatase	P11511	CYP19A1	503
Vascular endothelial growth factor receptor 2	P35968	KDR	1356
Serine/threonine-protein kinase B-raf	P15056	BRAF	766
3-phosphoinositide-dependent protein kinase 1	O15530	PDPK1	556
Sex hormone-binding globulin	P04278	SHBG	402
Mineralocorticoid receptor	P08235	NR3C2	984
SEC14-like protein 2	O76054	SEC14L2	403
Aldo-keto reductase family 1 member C3	P42330	AKR1C3	323
Dipeptidyl peptidase 4	P27487	DPP4	766
Phenylethanolamine N-methyltransferase	P11086	PNMT	282
Disintegrin and metalloproteinase domain-containing protein 17	P78536	ADAM17	824
Tyrosine-protein phosphatase non-receptor type 11	Q06124	PTPN11	593
Retinoic acid receptor RXR-alpha	P19793	RXRA	462
Histone deacetylase 8	Q9BY41	HDAC8	377
Glutathione reductase, mitochondrial	P00390	GSR	522
Phosphatidylinositol 4,5-bisphosphate 3-kinase catalytic subunit gamma isoform	P48736	PIK3CG	1102
Cyclin-dependent kinase 6	Q00534	CDK6	326
Estrogen-related receptor gamma	P62508	ESRRG	458
Sorbitol dehydrogenase	Q00796	SORD	357
Nitric oxide synthase, endothelial	P29474	NOS3	1203
Cyclin-dependent kinase 5 activator 1	Q15078	CDK5R1	307
Hepatocyte growth factor receptor	P08581	MET	1390
Annexin A5	P08758	ANXA5	320
Corticosteroid 11-beta-dehydrogenase isozyme 1	P28845	HSD11B1	292
Death-associated protein kinase 1	P53355	DAPK1	1430
