# Supplementary material for: Uncovering the molecular mechanism of Gynostemma pentaphyllum (Thunb.) Makino against breast cancer using network pharmacology and molecular docking
Source: Medicine (Baltimore). 2022 Dec 9;101(49):e32165. doi: 10.1097/MD.0000000000032165 (PMC9750687; doi:10.1097/MD.0000000000032165)
Supplement: Supplementary file 3 [file medi-101-e32165-s003.pdf]

Table S3 BC related targets in TTD and Drugbank database

BC related targets in TTD	BC related targets in Drugbank	BC related targets in TTD and Drugbank
**Uniprot ID**		
P00533	O15438	P00533
P11362	O15440	P11362
Q9HBH1	Q92887	Q9HBH1
P00915	O15244	P00915
P00918	O15431	P00918
P22748	O15432	P22748
Q13547	O95255	Q13547
P21802	P35670	P21802
P03372	Q04656	P03372
P04626	P35354	P04626
P06213	P05164	P06213
O60674	P47989	O60674
P16234	P9WJI5	P16234
P12931	P11712	P12931
P42345	P20813	P42345
P00519	P06276	P00519
P35968	Q02928	P35968
P10275	Q9UNQ0	P10275
P11511	P30711	P11511
P12461	P04731	P12461
P23280	P02795	P23280
O43570	P00441	O43570
Q9ULX7	P09211	Q9ULX7
P11802	P15559	P11802
Q00534	P09488	Q00534
Q92731	P29372	Q92731
P04150	P01023	P04150
P07900	P02768	P07900
P08069	P02787	P08069
P32245	O00244	P32245
Q99720	P03372	Q99720
P42336	P08684	P42336
O00329	P05177	O00329
P48736	P21964	P48736
P08581	O15439	P08581
P15538	P33527	P15538
P05093	P46721	P05093
P08842	Q14973	P08842
P00749	Q63ZE4	P00749

P07550	Q8TCC7	P07550
P10415	Q9NYB5	P10415
Q9UNQ0	Q9Y6L6	Q9UNQ0
P61073	O94956	P61073
P11387	P08183	P11387
Q02880	Q4U2R8	Q02880
P14416	Q86UW1	P14416
P21917	Q86UW2	P21917
Q96RI1	Q96BD0	Q96RI1
P11229	Q96J66	P11229
P06401	Q9NPD5	P06401
P16083	Q9NSA0	P16083
P48443	Q9UIG8	P48443
O14746	Q9UNQ0	O14746
P30530	P05543	P30530
O00418	P04278	O00418
P09919	P02768	P09919
P04141	Q92731	P04141
P03366	P11712	P03366
P20839	P33261	P20839
P38484	P11509	P38484
P29460	P33260	P29460
P22888	P10632	P22888
P36544	P20815	P36544
Q9NZQ7	O75469	Q9NZQ7
Q15116	P11388	Q15116
P07766	P33527	P07766
P15692	P16662	P15692
P12268	P47712	P12268
P11388	P04818	P11388
P01148	P19971	P01148
P9WP30	Q12882	P9WP30
P40198	Q16831	P40198
Q9NQ25	O95045	Q9NQ25
P03420	P11509	P03420
Q16790	P42898	Q16790
P22607	P11172	P22607
P31749	Q06203	P31749
P78536	Q9Y694	P78536
Q02763	Q99808	Q02763
O14965	O15439	O14965
P06493	P05543	P06493
P14555	P14324	P14555
Q9Y243	O95749	Q9Y243

Q15389	P00374	Q15389
O15123	Q06278	O15123
P21860	Q4U2R8	P21860
O14672	Q5T3U5	O14672
P42338	Q8TCC7	P42338
P18031	Q92887	P18031
P61812	P08183	P61812
P06731	P46721	P06731
P10909	P53985	P10909
P30550	Q9NSA0	P30550
Q9HBH9	Q9UIG8	Q9HBH9
Q9BUB5	P52209	Q9BUB5
P42356	Q96NT5	P42356
P23510	Q05932	P23510
P28336	Q92820	P28336
P32247	P04818	P32247
P32970	P31939	P32970
Q96BI3	Q6ZQN7	Q96BI3
Q8WW43	P41440	Q8WW43
Q9NZ42	P15328	Q9NZ42
Q92542	P08684	Q92542
P49768	P14207	P49768
P08238	P46059	P08238
Q14680	Q7Z2H8	Q14680
Q13421	P16435	Q13421
P15941	P31513	P15941
Q01973	P00533	Q01973
P17844	P02763	P17844
P17813	P19652	P17813
O94925	P14324	O94925
Q9UI32	O95749	Q9UI32
Q12988	P42574	Q12988
Q13641	P55211	Q13641
Q13433	P10635	Q13433
P09693	P10632	P09693
P04155	P23141	P04155
P16422	P20815	P16422
P01344	P04798	P01344
O43490	Q16678	O43490
Q9BPZ7	P33261	Q9BPZ7
E9PEK4	Q01740	E9PEK4
O15392	P31513	O15392
Q96CA5	P24462	Q96CA5
Q13075	P11511	Q13075

Q13489	P05181	Q13489
Q13490	Q9HAW8	Q13490
P98170	P50225	P98170
P16435	Q15125	P16435
P04792	P17252	P04792
Q9Y6Q9	P05771	Q9Y6Q9
P26447	Q05655	P26447
P51587	Q02156	P51587
Q99500	P05129	Q99500
O76070	P41743	O76070
Q5FBB7	Q04759	Q5FBB7
	Q05513	O15438
	P10275	O15440
	Q12809	Q92887
	O75469	O15244
	P62508	O15431
	P04278	O15432
	O95342	O95255
	Q06520	P35670
	O75795	Q04656
	P49888	P35354
	O95477	P05164
	P45983	P47989
	P22309	P9WJI5
	P18405	P11712
	P31213	P20813
	Q9H8P0	P06276
	P04626	Q02928
	Q96FL8	P30711
	Q86VL8	P04731
	Q9HB55	P02795
	P20815	P00441
	P24462	P09211
	P21860	P15559
	Q12882	P09488
	Q9NWZ5	P29372
	P10186	P01023
	Q8X444	P02768
	P0AA43	P02787
	Q5SKC5	O00244
	P77836	P08684
	P31645	P05177
	P23975	P21964
	Q01959	O15439

P33527
P46721
Q14973
Q63ZE4
Q8TCC7
Q9NYB5
Q9Y6L6
O94956
P08183
Q4U2R8
Q86UW1
Q86UW2
Q96BD0
Q96J66
Q9NPD5
Q9NSA0
Q9UIG8
Q9UNQ0
P05543
P04278
P02768
Q92731
P11712
P33261
P11509
P33260
P10632
P20815
O75469
P11388
P33527
P16662
P47712
P04818
P19971
Q12882
Q16831
O95045
P11509
P42898
P11172
Q06203
Q9Y694
Q99808

O15439
P05543
P14324
O95749
P00374
Q06278
Q4U2R8
Q5T3U5
Q8TCC7
Q92887
P08183
P46721
P53985
Q9NSA0
Q9UIG8
P52209
Q96NT5
Q05932
Q92820
P04818
P31939
Q6ZQN7
P41440
P15328
P08684
P14207
P46059
Q7Z2H8
P31513
P00533
P02763
P19652
P14324
O95749
P42574
P55211
P10635
P10632
P23141
P20815
P04798
Q16678
P33261
Q01740

P31513
P24462
P05181
Q9HAW8
P50225
Q15125
P17252
P05771
Q05655
Q02156
P05129
P41743
Q04759
Q05513
Q12809
O75469
P62508
P04278
O95342
Q06520
O75795
P49888
O95477
P45983
P22309
P18405
P31213
Q9H8P0
Q96FL8
Q86VL8
Q9HB55
P20815
P24462
P21860
Q12882
Q9NWZ5
P10186
Q8X444
P0AA43
Q5SKC5
P77836
P31645
P23975
Q01959
