# Supplementary material for: Uncovering the molecular mechanism of Gynostemma pentaphyllum (Thunb.) Makino against breast cancer using network pharmacology and molecular docking
Source: Medicine (Baltimore). 2022 Dec 9;101(49):e32165. doi: 10.1097/MD.0000000000032165 (PMC9750687; doi:10.1097/MD.0000000000032165)
Supplement: Supplementary file 4 [file medi-101-e32165-s004.pdf]

Table S4 The detailed information of common targets between BC and GP

Protein names	Uniprot ID	Gene names	Length
Epidermal growth factor receptor	P00533	EGFR	1210
Carbonic anhydrase 1	P00915	CA1	261
Carbonic anhydrase 2	P00918	CA2	260
Estrogen receptor	P03372	ESR1	595
Vascular endothelial growth factor receptor 2	P35968	KDR	1356
Androgen receptor	P10275	AR	920
Aromatase	P11511	CYP19A1	503
Carbonic anhydrase 12	O43570	CA12	354
Cyclin-dependent kinase 6	Q00534	CDK6	326
Heat shock protein HSP 90-alpha	P07900	HSP90AA1	732
Phosphatidylinositol 4,5-bisphosphate 3-kinase catalytic subunit gamma isoform	P48736	PIK3CG	1102
Hepatocyte growth factor receptor	P08581	MET	1390
Steryl-sulfatase	P08842	STS	583
Urokinase-type plasminogen activator	P00749	PLAU	431
Progesterone receptor	P06401	PGR	933
Disintegrin and metalloproteinase domain-containing protein 17	P78536	ADAM17	824
Aurora kinase A	O14965	AURKA	403
Cholinesterase	P06276	BCHE	602
Glutathione S-transferase P	P09211	GSTP1	210
Albumin	P02768	ALB	609
Dihydrofolate reductase	P00374	DHFR	187
Liver carboxylesterase 1	P23141	CES1	567
Estrogen-related receptor gamma	P62508	ESRRG	458
Sex hormone-binding globulin	P04278	SHBG	402
Sulfotransferase 2A1	Q06520	SULT2A1	285
Mitogen-activated protein kinase 8	P45983	MAPK8	427
