# Supplementary material for: Uncovering the molecular mechanism of Gynostemma pentaphyllum (Thunb.) Makino against breast cancer using network pharmacology and molecular docking
Source: Medicine (Baltimore). 2022 Dec 9;101(49):e32165. doi: 10.1097/MD.0000000000032165 (PMC9750687; doi:10.1097/MD.0000000000032165)
Supplement: Supplementary file 5 [file medi-101-e32165-s005.pdf]

Table. S5 Go and KEGG analysis of potential targets related to occurrence and development of BC

Description	Target number	–Log *P*	Category
GO: 0048545: response to steroid hormone	8	9.2794716	Biological Processes
GO: 0060745: mammary gland branching involved in pregnancy	3	7.7793395	
GO: 0071383: cellular response to steroid hormone stimulus	6	7.5623154	
GO: 0006730: one-carbon metabolic process	4	7.5185832	
GO: 0071396: cellular response to lipid	8	7.4877476	
GO: 0071407: cellular response to organic cyclic compound	8	7.4299829	
GO: 0050673: epithelial cell proliferation	7	7.3017361	
GO: 0048754: branching morphogenesis of an epithelial tube	5	6.8747361	
GO: 0009611: response to wounding	7	6.6991636	
GO: 0061138: morphogenesis of a branching epithelium	5	6.4739613	
GO: 0005788: endoplasmic reticulum lumen	5	5.0494605	Cell Components
GO: 0031983: vesicle lumen	4	3.6613574	
GO: 0001726: ruffle	3	3.2294203	
GO: 0097489: multivesicular body, internal vesicle lumen	1	3.0346593	
GO: 0009925: basal plasma membrane	3	2.7814867	
GO: 0097057: TRAF2-GSTP1 complex	1	2.7338221	
GO: 0070435: Shc-EGFR complex	1	2.7338221	
GO: 0045178: basal part of cell	3	2.6928932	
GO: 0042585: germinal vesicle	1	2.5579237	
GO: 0005943: phosphatidylinositol 3-kinase complex, class IA	1	2.5579237	
GO :0005496: steroid binding	5	7.8923684	Molecular Function
GO: 0030235: nitric-oxide synthase regulator activity	3	7.4602195	
GO: 0004879: nuclear receptor activity	4	7.1321504	
GO: 0098531: ligand-activated transcription factor activity	4	7.1321504	
GO: 0004089: carbonate dehydratase activity	3	6.7278064	
GO: 0051117: ATPase binding	4	6.1852243	

GO: 0004672: protein kinase activity	7	6.1587588	
GO: 0003707: steroid hormone receptor activity	3	6.0815858	
GO: 0001223: transcription coactivator binding	3	5.6940731	
GO: 0016773: phosphotransferase activity, alcohol group as acceptor	7	5.66357	
hsa05200: Pathways in cancer	7	9.4954596	KEGG pathway
hsa05120: Epithelial cell signaling in Helicobacter pylori infection	4	7.5148659	
hsa05215: Prostate cancer	4	7.0805389	
hsa00910: Nitrogen metabolism	3	6.55374856	
hsa04151: PI3K-Akt signaling pathway	6	6.44272242	
hsa05205: Proteoglycans in cancer	5	6.20190563	
hsa04915: Estrogen signaling pathway	4	6.1880721	
hsa05418: Fluid shear stress and atherosclerosis	4	6.15249824	
hsa05224: Breast cancer	4	5.99567255	
hsa04510: Focal adhesion	4	5.63987053	
hsa05212: Pancreatic cancer	3	5.39760342	
hsa05218: Melanoma	3	5.29871952	
hsa04014: Ras signaling pathway	4	5.18645276	
hsa01521: EGFR tyrosine kinase inhibitor resistance	3	5.12121336	
hsa01522: Endocrine resistance	3	4.86646822	
hsa04914: Progesterone-mediated oocyte maturation	3	4.86646822	
hsa05160: Hepatitis C	3	4.13297939	
hsa05206: MicroRNAs in cancer	4	4.06158843	
hsa04114: Oocyte meiosis	3	3.91823698	
hsa04015: Rap1 signaling pathway	3	3.7483441	
